# Digital Applications in the Fabrication of Obturators in Maxillectomy Defects: A Systematic Review

**DOI:** 10.7759/cureus.70479

**Published:** 2024-09-30

**Authors:** Arpita Paul, Pankaj Dhawan, Neha Jain

**Affiliations:** 1 Department of Prosthodontics, Manav Rachna Dental College, Faridabad, IND; 2 Department of Prosthodontics and Implantology, Manav Rachna Dental College, Faridabad, IND

**Keywords:** digitalization, maxillectomy defect, maxillofacial prosthesis, obturators, prosthetic dentistry, prosthodontic rehabilitation and maxillofacial prosthesis

## Abstract

The traditional approach of rehabilitating maxillofacial defects, as in the case of patients with maxillectomy, is exceedingly complex, tedious, technique-dependent, as well as extremely lengthy because it calls for several clinical appointments. The field of maxillofacial prosthodontics has benefited greatly from the introduction of digital technologies in the modern era. This systematic review was conducted to analyze the outcomes of applications of digital technology for the fabrication of obturators in patients with maxillectomy defects.

The PICOS (Population, Intervention, Comparison, Outcomes, and Study) inclusion criteria were employed in the systematic review, which focused on the query “How effective are obturators fabricated using digital techniques?” An electronic search of databases from 2017 to 2024 (Google Scholar, PubMed, Scopus) based on the predetermined eligibility criteria yielded 13 case reports and observational studies. The qualitative methodological assessment of the included studies was done based on the NIH quality assessment tool that provided criteria for special considerations in evaluating case reports and observational studies. The objective of the review was to analyze the application of various digital techniques for the fabrication of obturators in maxillectomy patients as well as to determine if a fully digital workflow is a viable option for effective fabrication of the same. The main outcome of the review was that the application of digital workflows in the fabrication of obturators was found to reduce working time, minimize material consumption, and enhance patient comfort and acceptance.

## Introduction and background

Maxillofacial defects, whether acquired or congenital, tend to significantly impair the esthetics and functional ability of the patients. The World Health Organization (WHO) has stated that orofacial cancer, a major contributor to maxillofacial defects, is most common in the Indian subcontinent [1]. The common treatment options for the restoration of these defects include reconstruction surgery, radiation therapy, chemotherapy, and combination therapies. Prosthodontists also play a pivotal role in the rehabilitation of maxillofacial defects. The traditional approach of rehabilitating such defects, as in case the of patients with maxillectomy, can be exceedingly complex, tedious, technique-dependent, as well as extremely lengthy since it requires several clinical appointments.

The field of maxillofacial prosthodontics has benefited greatly from the introduction of digital technologies in the modern era. When compared to the conventional procedure, it has significantly enhanced both the functional and aesthetic results, and it also has the added benefit of achieving them in less time [2]. Maxillofacial prosthesis construction has been demonstrated to benefit from various digital approaches, including intraoral scanning, 3D printing, and cone beam CT (CBCT). Obturators are being designed and printed digitally. To fabricate an obturator prosthesis, the traditional approach can be integrated with 3D printing, and a definitive prosthesis could be easily designed and printed with CAD/CAM (computer-aided design and computer-aided manufacturing) [3].

Due to several reasons, such as particular anatomic features and tumor recurrences, numerous patients continue to receive obturator prostheses through conventional methods, although surgical reconstruction happens to be the preferred mode of treatment for maxillectomy defects, which is cumbersome for the patient as well as the clinician. Therefore, with the advent of digital techniques pertaining to dentistry, the integration of different digital processes in the manufacture of maxillofacial prostheses has made the procedure more acceptable to these patients. Although some old methods of constructing obturator prostheses have been replaced by digital approaches, there is still a dearth of literature demonstrating a fully digital workflow for this purpose [4-7].

Therefore, we conducted this review to address the lacunae in the literature about the application and efficiency of digital techniques in the fabrication of obturators. We believe it will also aid the prosthodontist in making evidence-based clinical decisions concerning patients with maxillectomy. Hence, the objective of the present study was to review and analyze the digital technologies used for the fabrication of obturator prostheses in the prosthetic rehabilitation of maxillectomy defects and their efficacy.

## Review

Materials and methods

In conducting this review, we adhered to Preferred Reporting Items for Systematic Reviews and Meta-analyses (PRISMA) guidelines. The protocol had been registered on PROSPERO (International Prospective Register of Systematic Reviews) with the registration number CRD42024561626. The PICOS (Population, Intervention, Comparison, Outcomes, and Study) criteria employed in the systematic review included population (P) as “patients with maxillectomy defects,” intervention (I) as “applications of digital techniques in obturator fabrication,” control (C) as “digital technique vs. conventional method of fabrication of obturators,” and outcome (O) as “the efficacy of obturator prostheses fabricated using digital technology based on patient comfort and ease of fabrication.” We focused on addressing the following aspect: “Applying digital technology and assessing the efficacy of obturators fabricated using digital techniques.”

The inclusion criteria consisted of case reports, case series, observational studies, and randomized controlled trials on the fabrication of obturators in patients with maxillectomy defects using digital techniques from 2017 to 2024. Systematic reviews or literature reviews, articles in any language other than English, and articles where conventional methods have been used for obturator fabrication were excluded from the present study.

Systematic search strategy

An electronic search was performed on the databases PubMed, Scopus, and Google Scholar, spanning the period from 2017 to 2024. A search strategy consisting of combinations of controlled terms (MeSH) was applied. The following search strategy was used for all the databases (PubMed, Scopus, and Google Scholar), which included: MeSH terms: "obturator" AND "digital workflow" AND "fabrication" OR "maxillectomy" OR ''obturator" OR "maxillofacial prosthesis."

Two reviewers (AP and NJ) meticulously examined the articles from the obtained results by their titles and abstracts in the first two stages, respectively. After the removal of duplicates, studies that did not satisfy the inclusion criteria of the present study were excluded. Then, the full text of articles identified from the screening of titles and abstracts was reviewed by two reviewers (AP and NJ). Any conflict that arose during the screening of articles was resolved by a third reviewer (PD).

Study selection

**Figure 1:**
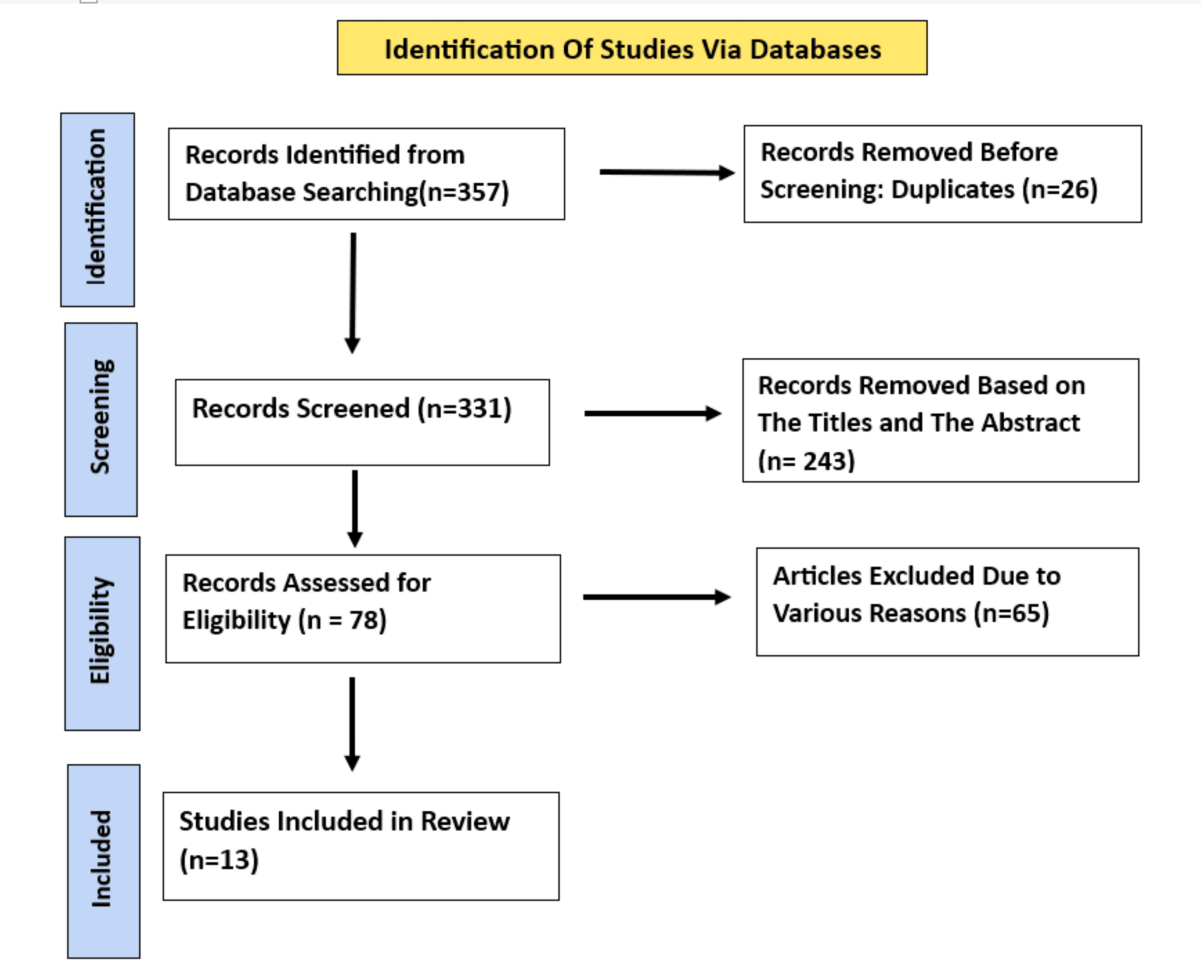
PRISMA flow diagram depicting the study selection PRISMA: Preferred Reporting Items for Systematic Reviews and Meta-Analyses

The initial number of identified articles was 357, out of which 26 articles were found to be duplicates, which were then eliminated, and the remaining 331 articles underwent title and abstract screening. Following this, 243 articles were excluded, leaving 78 articles, of which 65 articles were excluded as they did not meet the inclusion criteria. Finally, a total of 13 articles were included in the study as they met the inclusion criteria, out of which nine were case reports and four were observational studies. The study selection has been done based on PRISMA guidelines (Figure 1).

Data collection process and data items

A Microsoft Excel spreadsheet was utilized to document all the assimilated data from the included studies. The data collection table included the various observational studies and case reports wherein obturators have been fabricated using digital techniques.

Risk of bias/quality assessment

All the included studies were assessed using a criterion given by NIH for quality assessment of case reports and observational studies [8]. The criteria consisted of nine questions for case reports/series, while it consisted of 14 questions for observational studies. Quality was rated as poor (0-4 out of 14 questions; 0-2 out of nine questions), fair (5-10 out of 14 questions; 3-6 out of nine questions), or good (11-14 out of 14 questions; 6-9 out of nine questions).

According to qualitative assessment of bias using the NIH tool for quality assessment for case reports and observational studies, the thirteen studies [4, 9-20] included in the review were assessed, out of which five case reports and one observational study showed low risk of bias, whereas four case reports and three observational studies showed moderate risk of bias (Tables 1-2).

**Table 1:** Quality assessment for case reports (NIH) NA: not applicable; NR: not reported

	Park et al. (2017) [9]	Tasopoulos et al. (2018) [11]	Michelinakes et al. (2018) [12]	Tasopoulos et al. (2020) [13]	Ye et al. (2021) [4]	Gupta et al (2023) [16]	Ogami et al. (2023) [18]	Shahid et al. (2023) [19]	Handel et al. (2024) [20]
1. Was the study question or objective clearly stated?	Yes	Yes	Yes	Yes	Yes	Yes	Yes	Yes	Yes
2. Was the study population clearly and fully described, including a case definition?	Yes	Yes	Yes	Yes	Yes	Yes	Yes	Yes	Yes
3. Were the cases consecutive?	NA	NR	NR	NA	NR	NA	NA	NA	NA
4. Were the subjects comparable?	NA	NR	Yes	Yes	NR	NA	NA	Yes	NA
5. Was the intervention clearly described?	Yes	Yes	Yes	Yes	Yes	Yes	Yes	Yes	Yes
6. Were the outcome measures clearly defined, valid, reliable, and implemented consistently across all study participants?	Yes	Yes	Yes	Yes	Yes	Yes	Yes	Yes	Yes
7. Was the length of follow-up adequate?	No	Yes	Yes	Yes	Yes	Yes	Yes	Yes	Yes
8. Were the statistical methods well-described?	NR	NR	NR	NR	NR	NA	Yes	Yes	Yes
9. Were the results well-described?	Yes	Yes	Yes	Yes	Yes	Yes	Yes	Yes	Yes
Quality rating (good, fair, poor)	Fair	Fair	Good	Good	Fair	Fair	Good	Good	Good

**Table 2:** Quality assessment for observational studies (NIH) NA: not applicable; NR: not reported

	Rodney et al. (2017) [10]	Akram et al. (2020) [14]	Dartaguiette et al. (2021) [15]	Alanezi et al. (2023) [17]
1. Was the research question or objective in this paper clearly stated?	Yes	Yes	Yes	Yes
2. Was the study population clearly specified and defined?	Yes	Yes	Yes	NA
3. Was the participation rate of eligible persons at least 50%?	NA	Yes	NR	NA
4. Were all the subjects selected or recruited from the same or similar populations (including the same time period)? Were inclusion and exclusion criteria for being in the study prespecified and applied uniformly to all participants?	NA	Yes	Yes	NA
5. Was a sample size justification, power description, or variance and effect estimates provided?	NA	NR	No	Yes
6. For the analyses in this paper, were the exposure(s) of interest measured prior to the outcome(s) being measured?	No	No	No	No
7. Was the time frame sufficient so that one could reasonably expect to see an association between exposure and outcome if it existed?	Yes	Yes	Yes	Yes
8. For exposures that can vary in amount or level, did the study examine different levels of the exposure as related to the outcome (e.g., categories of exposure, or exposure measured as a continuous variable)?	Yes	Yes	No	NA
9. Were the exposure measures (independent variables) clearly defined, valid, reliable, and implemented consistently across all study participants?	Yes	Yes	Yes	Yes
10. Was the exposure(s) assessed more than once over time?	No	No	No	No
11. Were the outcome measures (dependent variables) clearly defined, valid, reliable, and implemented consistently across all study participants?	Yes	Yes	Yes	Yes
12. Were the outcome assessors blinded to the exposure status of participants?	NR	Yes	NR	NR
13. Was the loss to follow-up after baseline 20% or less?	NR	Yes	NR	NR
14. Were key potential confounding variables measured and adjusted statistically for their impact on the relationship between exposure(s) and outcome(s)?	No	Yes	No	Yes
Quality rating (Good, fair, or poor)	Fair	Good	Fair	Fair

Results

The outcomes and results of the studies included in this review are described in Table 3.

**Table 3:** Details of the individual studies included in the review 3D: three-dimensional; CAD/CAM: computer-aided design and computer-aided manufacturing; CT: computed tomography

Sl. no.	Year	Author	Study design	Type of workflow	Outcome	Results
1	2017	Park et al. [9]	Case report	Fabrication of a maxillary obturator for a patient with partial maxillectomy, using an intra-oral digital impression	Assessment of whether using intraoral scanners for obturator fabrication can be a viable alternative to the conventional method	The maxillary obturator fabricated using intra-oral digital impression showed acceptable short-term treatment outcomes
2	2017	Rodney et al. [10]	Observational study	Use of CT data to digitally fabricate surgical obturators	This method precludes the need for an impression appointment and enhances patient comfort	The technique described in this study allows multiple versions of the surgical obturator to be fabricated with relative ease to allow for unpredictable surgical margins created during the maxillectomy surgery
3	2018	Tasopoulos et al. [11]	Case report	Fabrication of an interim obturator using 3D printing	Eliminating discomfort to maxillectomy patients during conventional impression technique	The 3D printing technology used to fabricate the interim maxillary obturator eliminated the need for uncomfortable conventional impression techniques, facilitating the shortening of treatment time as well
4	2018	Michelinakis et al. [12]	Case report	Use of intra-oral scanner and CAD/CAM for fabrication of obturator prosthesis in patient with hemi-maxillectomy	To eliminate the need for conventional impressions leading to a reduction in the number of appointments and increase patient comfort	The intra-oral scanning of the hemi-maxillectomy defect and implementation of CAD/CAM techniques for obturator fabrication serve as a viable option for less tissue irritation and more patient comfort
5	2020	Tasopoulos et al. [13]	Case report	Fabrication of a PEEK maxillary hollow-bulb obturator using intra-oral scanning, 3D printing, and CAD/CAM	The obturator fabricated with PEEK was to be more biocompatible and lightweight	The procedure described, resulted in an accurate two-piece hollow-bulb obturator while avoiding the discomfort associated with analog impressions
6	2020	Akram et al. [14]	Observational study	Evaluation of dimensional accuracy and intimacy of single-piece hollow-bulb obturators fabricated using CAD/CAM additive manufacturing with that of the ones fabricated with heat-cured resin	Additive manufacturing as a viable alternative to conventional technique for direct construction of hollow-bulb obturators	CAD/CAM obturators showed lower misfit values when compared to the heat-cured ones
7	2021	Dartaguiette et al. [15]	Observational study	Personalized and lightweight surgical obturator fabricated by digital simulation of the maxillectomy defect and designing of obturator using open-source software and 3D printing	The immediate surgical obturator fabricated by this method was a biocompatible, personalized, and lightweight approach for the patient	The immediate surgical obturator fabricated, adapted to the maxillectomy defect with the requirement of a minimal amount of relining material
8	2021	Ye et al. [4]	Case report	Fully digital workflow for design and manufacture of obturator for maxillectomy defects	Assessment of accuracy of obturator fabricated using a fully digital workflow	The fully digital technique allowed efficient recording of the cavity of the defect accurately, and more convenient fabrication of a hollow-bulb obturator without patient discomfort
9	2023	Gupta et al. [16]	Case report	Digital technique for fabrication of surgical obturator with decreased mouth opening in a patient with a hemi-maxillectomy defect	Fabrication of the obturator using intraoral scanning technology is a viable option as compared to the conventional method	Intraoral scanning of hemi-maxillectomy defects and implementation of CAD/CAM for obturator fabrication served as a viable option for less tissue irritation and more patient comfort.
10	2023	Alanezi et al. [17]	Observational study	Fabrication of 3D-printed laboratory models of maxillectomy defects and comparing digital impressions with conventional techniques in terms of dimensional accuracy and time consumed	Development and comparison of conventional and 3D-printed models of maxillectomy defects	The time consumed for recording the impression using an intraoral scanner was significantly less than that of the conventional impression technique.
11	2023	Ogami et al. [18]	Case report	Optical impressions and occlusal records were taken and a maxillary obturator was fabricated using a CAD/CAM system	The use of digital data and optical impressions instead of conventional ones shortened the duration of fabrication of the obturator as well as prevented patient discomfort	After two years of obturator fabrication, the patient as well as the prosthesis were found to be in good condition
12	2024	Shahid et al. [19]	Case report	Use of digital design and printing for fabrication of an interim obturator for a patient with a history of maxillary sinus squamous cell carcinoma and limited mouth opening.	Minimally invasive treatment approach for fabrication of interim obturator and restoring patient function	The interim obturator prosthesis fabricated using the digital approach evades patient discomfort and serves as a viable option for restoring oral function quickly
13	2024	Handel et al. [20]	Case report	Utilizing CT scan data to generate 3D models for the fabrication of surgical obturator as making conventional impression would interfere in the healing process of maxillectomy defect	Practicality and efficiency for prosthodontists for fabrication of surgical obturators	Efficient and reliable method for fabricating a surgical obturator in the absence of conventional impressions

Discussion

Rehabilitation of maxillectomy defects can be challenging. Maxillofacial defects, such as the ones in the case of patients who underwent maxillectomy, can be caused by surgical resection of malignant lesions, congenital abnormalities, as well as trauma. Such defects can lead to oro-nasal communication, which in turn can affect several functional aspects, including phonation, mastication, and deglutition. Such defects eventually impact the quality of life of the patients, subsequently also affecting their psychological status. These defects may be rehabilitated through surgical reconstruction or prosthodontic reconstruction, including the fabrication of obturator prostheses, depending on the clinical and physical condition of the patient. Therefore, prosthodontists play a crucial role in the rehabilitation of maxillectomy patients. Hence, meticulous knowledge of making precise impressions of defects in maxillectomy patients is extremely necessary for adequate prosthetic treatment, minimizing time for clinical appointments and providing greater patient comfort [6].

The traditional procedure for constructing an obturator prosthesis requires making an impression, including the defect of the maxillary arch, following a series of complex methods that are technique-sensitive and may lead to several complications as well as cause significant discomfort to the patient. These complications may include dislodgement of impression material into the cavity of the defect, allergic reactions toward a foreign body within a healing cavity, and secondary infections necessitating hospitalization. The physical cast models fabricated from conventional impression procedures may get frequently damaged, deteriorated, misplaced, or weathered, which warrants taking another set of impressions before definitive prostheses fabrication. These consequently create problems for both the patient as well as the prosthodontist, prolonging the duration of treatment and may also compromise the clinical success of the prosthetic rehabilitation. Recently, the introduction of intraoral scanners, 3D printing, and rapid prototyping in the field of prosthodontics has introduced several techniques through which these issues can be averted [7]. Although digital techniques are gaining popularity in the field of prosthodontics, further research and investigation are needed to assess the efficacy of various digital techniques in the fabrication of obturators and serve as a viable option for the same [5]. Therefore, to address this deficit of knowledge in the current literature, the current systematic review has been carried out.

Obturator prostheses fabricated using digital impression have been found to have shown acceptable short-term treatment outcomes, as suggested by Park et al. (2017), in a clinical case wherein they assessed whether using intraoral scanners for obturator fabrication in a patient with partial maxillectomy can be a viable alternative to conventional methods. They concluded that digital impressions can reduce patient discomfort and make impression-making easier [9].

CT data, when used to digitally fabricate surgical obturators, allows multiple versions of the obturator to be fabricated with relative ease and evades the circumstance of causing extreme discomfort to the patients. This also aligns with an observational study conducted by Rodney and Chicchon in 2017, where it was observed that using CT data to fabricate surgical obturators enhanced patient comfort and precluded the need for an impression appointment [10]. Tasopoulos et al. [11], in 2018, reported a case in which 3D-printing technology was used to fabricate the interim maxillary obturator, which in turn eliminated the need for uncomfortable conventional impression techniques, facilitating the shortening of treatment time as well. In the same year (2018), Michelinakis et al. reported a case in which they utilized intra-oral scanning of the hemi-maxillectomy defect in the patient and implementation of CAD/CAM techniques for obturator fabrication. They concluded that the use of the aforementioned digital techniques serves as a viable option for less tissue irritation and more patient comfort [12].

In a procedure where PEEK was used for the fabrication of a maxillary hollow-bulb obturator using intraoral scanning, 3D printing, and CAD/CAM by Tasopoulos et al. in 2020, it resulted in a lightweight and accurate two-piece hollow-bulb obturator while avoiding the discomfort associated with analog impressions [13]. Similarly, Akram et al. conducted a study in 2020 to evaluate the dimensional accuracy and intimacy of single-piece hollow-bulb obturators fabricated using CAD/CAM additive manufacturing with that of the ones fabricated with heat-cured resin. They found that CAD/CAM obturators showed lower misfit values when compared to the heat-cured ones and finally concluded that additive manufacturing can be a viable and successful alternative to conventional techniques for direct construction of hollow-bulb obturators [14].

A personalized and lightweight surgical obturator was fabricated using a digital simulation of the maxillectomy defect in a study conducted by Dartaguiette et al. in 2021. They designed the obturator using open-source software and 3D printing, which was reported to be a more comfortable and biocompatible approach to fabricating a surgical obturator [15]. In 2021, Ye et al. [4] reported a case wherein they assessed the accuracy of an obturator fabricated for maxillectomy patients using a fully digital workflow. The fully digital workflow used by them allowed efficient recording of the cavity of the defect accurately and more convenient fabrication of a hollow-bulb obturator without patient discomfort.

Fabrication of an obturator using intraoral scanning in a patient with hemi-maxillectomy defect and decreased mouth opening by Gupta et al. in 2023, and implementation of CAD/CAM for the same was reported to serve as a viable option for less tissue irritation and more patient comfort [16]. Alanezi et al. fabricated 3D-printed laboratory models of maxillectomy defects and compared digital impressions with conventional techniques in terms of dimensional accuracy and time consumed. They finally concluded that the time consumed for recording the impression using an intraoral scanner was significantly less than that of the conventional impression technique [17]. In 2023, Ogami et al. reported a case wherein a maxillary obturator was fabricated using optical impressions, occlusal records, and a CAD/CAM system. The use of digital data and optical impressions instead of conventional ones shortened the duration of fabrication of the obturator as well as prevented patient discomfort. After a two-year follow-up, they concluded that the patient as well as the prosthesis were in good condition [18].

The use of digital design and printing for the fabrication of an interim obturator for a patient with a history of maxillary sinus squamous cell carcinoma and limited mouth opening was described by Shahid et al. in 2024. They concluded that an interim obturator prosthesis fabricated using a digital technique evades patient discomfort and serves as a viable option for restoring oral function quickly as it is a minimally invasive approach [19]. In 2024, Handel et al. reported a case in which they utilized CT scan data of the patient to generate 3D models for the fabrication of a surgical obturator, as making a conventional impression would interfere with the healing process of a maxillectomy defect. It was found in their study to be an efficient and reliable method for fabricating a surgical obturator in the absence of conventional impressions [20].

After a qualitative assessment of the studies included in the current systematic review, it has been found that applying several digital techniques for the fabrication of obturators is a viable, more efficient, and reliable approach that evades immense discomfort to the patient as well as minimizes the working time. To the best of our knowledge, the present review, amidst its limitations, presents significant evidence of the advantages of using several digital applications for the fabrication of obturators, in patients with maxillectomy defects, which can aid a prosthodontist in making better clinical decisions while treating such patients.

Limitations of the study

The current systematic review has a few potential limitations because only limited databases have been accessed. Only limited studies on digital technologies that are used in the fabrication of obturators are available in the literature, and in the present study, only case reports and a few observational studies could be included, due to which meta-analysis was not possible to be performed. Case reports happen to be low in the hierarchy of evidence; hence, the recommendations and conclusions from this systematic review must be used judiciously. Despite this limitation, the present study was able to synthesize significant qualitative evidence from the literature to analyze the effectiveness of digital techniques that have been employed in obturator prostheses fabrication in patients with maxillectomy defects.

## Conclusions

The present systematic review includes case reports and observational studies that have applied various digital techniques in the fabrication of obturators for maxillectomy patients. The main outcome of the review was that digital techniques, when applied to the fabrication of obturators, were found to reduce working time, minimize material consumption, and enhance patient comfort and acceptance. Hence, we believe our findings may influence the work of prosthodontists in making evidence-based clinical decisions while planning the treatment of patients with maxillectomy defects and introducing some considerations regarding time efficiency and patient-focused outcomes by using a digital workflow in the fabrication of obturator prostheses for such patients.
